# Immunohistochemical Evaluation of p63, E-Cadherin, Collagen I and III Expression in Lower Limb Wound Healing under Honey

**DOI:** 10.1155/2011/239864

**Published:** 2011-03-31

**Authors:** Ananya Barui, Provas Banerjee, Raunak Kumar Das, Shyamal Kumar Basu, Santanu Dhara, Jyotirmoy Chatterjee

**Affiliations:** ^1^School of Medical Science & Technology, Indian Institute of Technology, Kharagpur 721302, India; ^2^Banerjee's Biomedical Research Foundation, Sainthia 731234, India

## Abstract

Honey is recognized traditionally for its medicinal properties and also appreciated as a topical healing agent for infected and noninfected wounds. This study evaluates impact of honey-based occlusive dressing on nonhealing (nonresponding to conventional antibiotics) traumatic lower limb wounds (*n* = 34) through clinicopathological and immunohistochemical (e.g., expression of p63, E-cadherin, and Collagen I and III) evaluations to enrich the scientific validation. Clinical findings noted the nonadherence of honey dressing with remarkable chemical debridement and healing progression within 11–15 days of postintervention. Histopathologically, in comparison to preintervention biopsies, the postintervention tissues of wound peripheries demonstrated gradual normalization of epithelial and connective tissue features with significant changes in p63^+^ epithelial cell population, reappearance of membranous E-cadherin (*P* < .0001), and optimum deposition of collagen I and III (*P* < .0001). Thus, the present study for the first time reports the impact of honey on vital protein expressions in epithelial and connective tissues during repair of nonhealing lower limb wounds.

## 1. Introduction

Wound healing involves complex and multifactorial biological processes with overlapping stages [1]. However, in nonhealing wounds, successive repairing stages are affected by varied pathological happening including infection [2–4], imbalance in extracellular matrix formation and degradation [5, 6], impaired re-epithelialization [7] and nutritional supply, adverse microenvironment, and repeated physical trauma [8]. So, healing interventions need to address these pathological variables to facilitate cellular and molecular events towards re-epithelialization, connective tissue formation, and maturation of regenerating tissues [9–11]. 

The re-epithelialization is orchestrated by keratinocyte proliferation and migration which are again correlated with expression of p63, a marker of undifferentiated proliferating cells [12] and development of stratified epithelial [13–15]. Therefore, the differential p63 expression in venous ulcers and other epidermal ulcers in comparison to healthy wounds are no doubt indicative in understanding the impaired healing [7, 16]. Nevertheless, membranous expression of E-cadherin, a calcium-dependent cell-surface glycoprotein responsible for cell-cell adhesion [17], is essential for establishing epithelial integrity [18, 19]. In the context of wound re-epithelialization, the involvement of E-cadherin especially in controlling cellular polarity [20], differentiation, growth, and migration [21] is crucial. Further, connective tissue formation during repair and to prevent scarring, optimum deposition of collagen I and III with their proper ratio is important [22, 23]. In particular, collagen I is responsible for higher tensile strength, whereas collagen III is predominantly found in early wound healing stages [9, 24, 25], and they maintain a particular ratio [26] in healthy skin. The geometrical arrangement of fibrillar collagen and their optimum ratio are vital during healing progression to maintain required crosslinked density and mechanical toughness [27, 28].

The lower extremity wound healing exhibits remarkable complexity due to negative impact of various anatomical and functional parameters as well as harsh effect of gravity on blood transportation. The taut foot skins are susceptible for frequent ulceration, as their terminal capillary plexus is exposed to various pressure gradients [29]. Evaluation of above-mentioned molecules in terms of their expression could be vital especially in assessing the impact of any healing interventions. 

Honey, being a natural nutritional reservoir containing various organic/inorganic substances including major amounts of carbohydrates along with lipids, amino acids, proteins, vitamin, bioelements [30, 31] with acidic pH (~4) possesses multidimensional prohealing effects for infected/noninfected wounds [32], finds application as a topical agent with optimal moisture retention capacity, debridement ability and anti-inflammatory effect [33–35]. However, deeper understanding is required for biological validation of its impact especially on vital cellular and molecular events related to important repair processes like re-epithelialization, subepithelial connective tissue formation of wounds to guide clinicians.

With these facts in mind, present study evaluates healing impact of honey as a topical agent in treatment of nonhealing traumatic lower limb wound (LLW) through immunohistochemical analysis of p63, E-cadherin, and collagen I and III in addition to routine histopathological and clinical observations.

## 2. Materials and Methods

### 2.1. Clinical Study

The patients (*n* = 34) of either gender (age = 16–65 years) with nonhealing lower extremity wounds (traumatic origin) with exudation of pus, foul smell, and necrotic tissues and nonresponding to conventional topical antibiotics were included under informed written consent. Ethical clearance was obtained from institutional ethical committee according to Helsinki declaration. Subsequently, physicochemically characterized honey- (ripe and dark amber colored honey with ~14% water content, ~4 pH and viscosity 4.16 Pas at 37°C, collected from bee keepers of greater Kolkata, India) based occlusive dressing (i.e., honey-soaked gauge followed by a layer of dry cotton tied with crepe elastic bandage) was applied on LLW. Redressing was performed with an interval of 24 hrs for initial 7-8 days having foul odor, exudation, and necrotic tissues in the wounds and with progression of healing interval increased to 48–72 hours. Clinically, pain, malodor, oedema, debridement, granulation tissue formation, and epithelialization were recorded.

### 2.2. Histopathological and Immunohistochemical Studies

Incisional biopsies from wound edge were collected from few cases (*n* = 21) under local anaesthesia (Xylocaine) before and after (i.e., 15th and 22nd day) honey dressing. The normal skin samples collected from superfluous tissues of surgical interventions.

#### 2.2.1. Tissue Processing

Biopsies fixed with 10% phosphate buffered formalin and processed for 4 *μ*m thick paraffin sections on poly-L-lysine (Cat. No. P 8920 Sigma-Aldrich, St. Louis, MO, USA) coated slides.

#### 2.2.2. Hematoxylin and Eosin (H and E) and Van Gieson's Staining (VG)

 Tissue sections stained with hematoxylin and eosin as well as VG [36].

#### 2.2.3. Immunohistochemistry

Tissue sections baked and deparaffinized then hydrated for antigen retrieval in 10 mM citrate buffer (pH 6.0) using EZ-Retriever System V.2 (BioGenex, San Ramon, California, USA) and immunostained with kit (i.e., Super Sensitive Polymer-HRP IHC Detection System Cat. no: QD400-60K BioGenex). Sections incubated with primary antibodies (antihuman p63 clone 4A4, Cat. no. AM418-5M, and anti-Collagen III clone HWD1.1, AM167-5M BioGenex; E-Cadherin, clone EP700Y, Cat. no. ab40772, and anti-Collagen I polyclonal, ab34710, Abcam, Cambridge, UK) p63 and Collagen III, whereas a dilution of 1 : 500 was used for E-cadherin and Collagen I. Primary antibody binding visualized using a horseradish peroxidase conjugated secondary antibody using the chromogen 3, 3′-diaminobenzidine (DAB) and counterstained with hematoxylin. Appropriate controls were put up to validate the experiments.

### 2.3. Microscopic Studies

 The histopathological and immunohistochemical assessments were performed using Zeiss Observer.Z1 Microscope (Carl Zeiss, Germany) under 20x (NA 0.8; pixel resolution 0.31) and 40x oil (NA 1.3; pixel resolution 0.16). The images were grabbed digitally by CCD camera (AxioCam MRC, Zeiss) at 1388 × 1040 pixels.

### 2.4. Semiquantitative Evaluation of Immunohistochemical Observations

 In this study, from each study class, that is, normal, pre- and postintervention (15th and 22nd days), 21 tissue sections were assessed. In this process, digital images from the respective study classes were selected randomly, and following analysis was performed with the help of expert histopathologists.

#### 2.4.1. Counting of p63^+^ Cells

 The p63^+^ epithelial cell population per microscopic field, throughout the epithelium, was counted under 20x objectives.

#### 2.4.2. Assessment of Intensity Variation for E-Cadherin, and Collagen I and III Expressions

**Figure 1:**
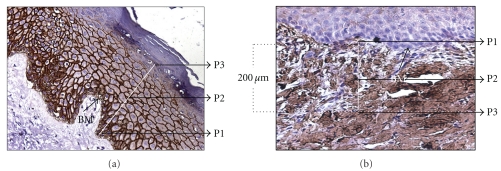
Schematic representation of the adopted method to generate intensity data for E-cadherin (a) and Collagens I and III (b). In both assessments, basement membrane (BM) was used as reference point. For E-cadherin (a), the P1, P2, and P3 along the white line represented three equidistant points in the epithelial expression path, while expression of collagen molecules in dermis (b) were assessed along 200 *μ*m (the white vertical line) below basement membrane at three equidistant points that is, P1, P2, P3.

 The expression intensities of E-Cadherin was measured along the expression path as per an intensity scoring scale (i.e., 0–10) at three equidistant points that is, P1, P2, and P3 of the epithelium (Figure 1(a)) using the software Axiovision (Version 4.7.2, Carl Zeiss, Germany). Further, to assess the cellular site specific E-cadherin expression and its overall distribution in the stratum basilaris and stratum spinosum in terms of color intensity (Table 2), a 10-point intensity scoring scale was used considering maximum membranous expression as “10” and maximum cytoplasmic expression as “0”. 

The intensity variations of the collagen I and III were measured at three equidistant study points, that is, P1, P2, and P3 (Figure 1(b)), within 200 *μ*m range below basement membrane using above-mentioned intensity [37] scoring scale. In this purpose, 30 random points were selected from photomicrograph of each study groups under 20x objective. Further, the ratio between Collagen I and III was measured from mean values of their intensities. All these semiquantative assessment were performed under guidance of expert histopathologist.

### 2.5. Statistical Evaluation

Independent sample “*t*”-test was applied for the analysis of p63 data. The E-cadherin and collagen expression intensity scores at different study points that is, P1, P2, and P3 were analyzed using analysis of variance (ANOVA).

## 3. Results

### 3.1. Healing of LLW under Topical Application of Honey

#### 3.1.1. Clinical Observations on LLW

**Figure 2:**
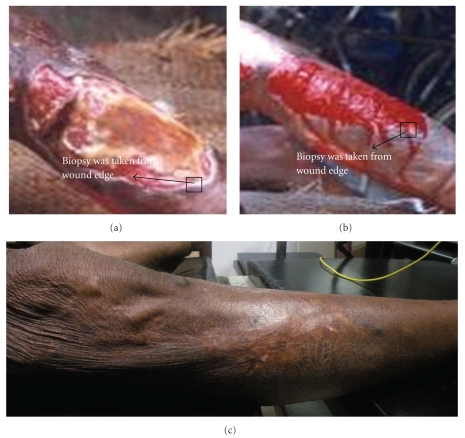
Representative photographs of lower limb wound showing healing under topical application of honey: (a) before application (b) 35 days, and (c) 180 days after application.

Clinically, the remarkable decrease of pain, oedema, and malodor was noted within 11–15 days of honey dressing. The granulation tissue formation and re-epithelialization were observed within 7–11 and 12–15 days, respectively, after intervention (Figure 2). Interestingly, there was no need of mechanical debridement of the necrotic tissues and nonadhesiveness of the dressing was significant in inhibiting further trauma to the wound bed.

### 3.2. Histopathological Observations

The microscopic observations under H and E staining revealed that on 15th and 22nd days after intervention, histological features were improved remarkably with the appearance of rete pegs and progressive maturation of the epithelial cells and connective tissue components. The VG studies depicted improvement in collagen population in respect to their density and distribution in achieving the random orientation like normal skin.

### 3.3. Immunohistochemical Findings

**Figure 3:**
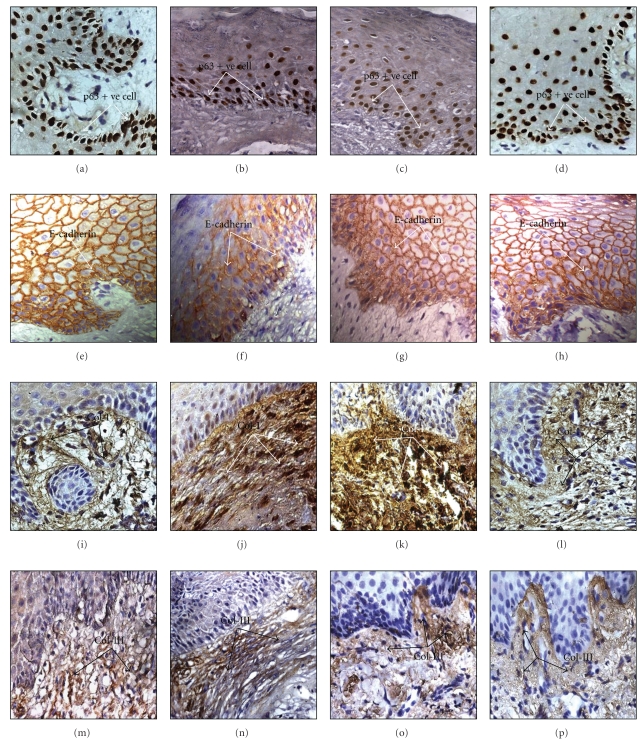
Immunohistochemical photomicrographs (40x oil, a-p) of skin biopsies (normal and leg wound periphery): (a)–(d) depicted expression of p63 in intact (a), before (b), after 15th days (c), and 22nd days (d) of topical intervention of honey: (e)–(h) demonstrated E-cadherin expression in different cellular layers of epithelium in normal (e) skin and in peripheral biopsies of before (f) and after said days of interventions (g), (h); (i)–(p) depicted expression of Collagen I (i)–(l) and Collagen III (m)–(p) in normal skin (i), (m), preintervention (j), (n) and in early (k), (o) and late (l), (p) periods of postintervention. The expression of p63 (b), E-cadherin (f), collagen I (j) and III (n) in flattened epithelium in preintervention stage and their changed expression status in normal and postintervention periods-p63^+^ nuclei distribution (a), (c), and (d), increased E-cadherin expression (e), (g), and (h), altered Collagen I (i), (k), and (l) and Collagen III (m), (o), and (p) density, orientation, and distribution.

In respect to p63^+^ cell population, differences were demonstrated between normal epithelium (Figure 3(a)) and wound bed margins before (Figure 3(b)) and after (Figures and 3(c) and 3(d)) honey dressing. In comparison to normal (p63^+^ ~ 90%), positive cell population was remarkably less (p63^+^ ~ 78.3%) in predressing biopsies, but the values moved towards normalcy on 15th (p63^+^ ~ 80.4%) and 22nd (p63^+^ ~ 86.8%) days of postintervention. 

In respect to E-cadherin expression (Figures 3(e)–3(h), there was remarkable difference between pre- (Figure 3(f)) and postapplication (Figures 3(g), and 3(h)) biopsies (Tables 1 and 2). In comparison to normal membranous expression of E-cadherin, it was mostly cytoplasmic (Figure 3(e)) and less intense (*P* < .0001) in epithelium of preintervention biopsies. However, in postintervention biopsies, the membranous expressions were significantly increased (*P* < .0001) mostly above the basal layers like normal skin (Figures 3(g), and 3(h)). Further, the assessment of E-cadherin expression in stratum basilaris and stratum spinosum depicted remarkable differences amongst pre- and postintervention biopsies and demonstrated the expression towards normalcy in stratum basilaris stratum spinosum on 20th day of postintervention (Table 2).

The collagen I and III expressions (Figures 3(i)–3(p)) in subepithelial connective tissue of preintervention biopsies showed significantly higher population density (*P* < .0001) with parallel orientation (Figures 3(j), and 3(n)) while in different postapplication biopsies both collagen types became less dense and distributed randomly (Figures 3(k), 3(l), and 3(o), 3(p)). Further, the ratio between collagen I and III (Table 1) was significantly high (1.57) in preintervention samples which was decreased in postintervention periods (i.e., 1.49 after 15 days and 1.31 after 22 days) towards the ratio of normal skin (1.25).

## 4. Discussion

During tissue repair the reconstitution of the epithelial barrier and connective tissue with optimum levels of collagen deposition is essential [38] and synchrony between the processes is vital in preventing anomaly in wound repair [39]. Hence, to understand topical impact of honey dressing on nonhealing wounds, present work focused on the evaluation of the immunohistochemical analysis on molecular expressions related to re-epithelization and connective tissue status to overcome inadequacy of routine clinicopathological gold standards. 

The reduction of pain, edema, malodor and debridement within reasonable time in LLW after such dressing as noted in this study signifies the anti-inflammatory, anti-bacterial and chemical debridement ability of acidic, viscous and hygroscopic honey [40–42]. These are corroborative with the previous findings that acidic pH of honey have influence on cellular, molecular cascades in wound bed to accelerate repair of nonhealing conditions [32] which shows tendency of acquiring alkaline pH [43, 44]. The nonadherence of dressing and optimum granulation tissue formation further indicated the prohealing efficacy of honey in minimizing re-dressing trauma and maintaining moist wound environment, crucial for healing [42].

These assumptions get corroboration from present histopathological and immunohistochemical findings. The H and E and VG studies demonstrated the characteristic change in epithelium and dermal collagen density and their distribution towards normalcy during postintervention periods. Whereas, immunohistochemical studies unveiled favorable modulation in expression of prime epithelial molecules like p63, E-cadherin and important members of dermal collagen like I and III (Figure 3) in the same periods. 

Interestingly, in preintervention samples, flattened epithelium (Figure 3(b)) with less p63^+^ cells demonstrated the abnormal reduction cellular proliferation in contrast to postintervention periods with increased p63^+^ cells and normal epithelial maturation features with the appearance of rete pegs (Figures 3(c) and 3(d)). The analysis of membranous expression of E-cadherin in postintervention biopsies (Figures 3(g) and 3(h)) in contrast to predominantly cytoplasmic expression in preintervention one (Tables 1 and 2) further indicated the restoration of coherent cell-cell adhesion, a good feature for healthy re-epithelialization [45–47]. In the context of such modulation in expression of Ca^+2^-dependent E-cadherin, the Ca^+2^ in honey may have significance [48–51].

The findings on collagen I and III (Figures 3(k), 3(l), and 3(o), 3(p)) in postintervention biopsies revealed gradual restoration of collagens towards normalcy (Figures 3(i) and 3(m)) in terms of their density distribution [21, 22] which were corroborative with the VG findings and also with less scarring. The Collagen I and III ratio analysis (Table 1) supported the notion that under honey healing, the regenerated skin achieved almost the normal ratio in respect to these major fibrillar molecular components [52]. 

Present approach allowed us to generate semiquantitative data on molecular expression for better interpretation of the immunohistochemical observations. The adopted quantification technique could differentiate the normal, pre- and postintervention biopsies in respect to their remarkable differences in molecular expression during progression of healing under honey. Further precision could be achieved in future studies by using fluorescent confocal imaging with spectral information and colour image processing. 

From the above discussion, it may be opined that the immunohistochemical findings not only demonstrated important molecular events in re-epithelialization and connective tissue formation in LLW before and after honey dressing for the first time but also corroborated with the clinical and histopathological findings to interpret the conversion of nonhealing wound into healing one after such intervention. Thus, these therapeutic impacts at the molecular levels that is the increased p63^+^ cell and membranous expression of E-cadherin as well as Collagen I and III deposition towards normalcy in the regenerated skin of LLW convincingly exhibited the molecular features to assess the wound healing progression under honey dressing. 

## 5. Conclusion

The honey with its diverse chemical constituents (organic and inorganic) provide therapeutic support to nonhealing lower limb wounds with minimum trauma during redressing and debridement as well as in healing without hyper-granulation and less scarring. Further, therapeutic potential has been demonstrated at molecular levels through immunohistochemical depiction of prime molecular expressions in wound biopsies. The gradual increase in p63^+^ cell population and membranous expression of E-cadherin pointed out the transformation of nonhealing wound into healing one and achievement of collagen I and III ratio towards normalcy in posttherapeutic periods indicated proper deposition of collagens in the regenerated skin during healing.

## Figures and Tables

**Table 1:** ANOVA in evaluating E-cadherin, Collagen I and III intensity differences within and between the study classes and assessment of collagen ratios.

Molecule of interest		Study groups											
		NP_1_	NP_2_	NP_3_	BP_1_	BP_2_	BP_3_	A_15_P_1_	A_15_P_2_	A_15_P_3_	A_22_P_1_	A_22_P_2_	A_22_P_3_
E-cadherin	Mean (Sd)	6.64 (0.99)	9.00 (1.04)	9.16 (0.94)	5.16 (1.18)	4.24 (1.45)	4.12 (1.99)	7.08 (1.35)	9.32 (1.14)	7.88 (1.13)	5.88 (1.20)	8.60 (1.47)	8.36 (1.29)
	*F* value within study gr.	50.39*			3.263*			21.90*			32.29*		
	*F* value between study gr.										89.07*		

Collagen I	Mean (Sd)	4.48 (1.16)	4.84 (1.14)	4.44 (1.73)	9.68 (0.63)	9.36 (0.7)	7.84 (0.72)	8.76 (0.93)	7.64 (1.44)	6.68 (1.38)	4.68 (1.07)	5.04 (1.54)	4.84 (2.12)
	*F* value within study gr.	0.64*			40.87*			16.85*			0.31*		
	*F* value between study gr.										173*		

Collagen III	Mean (Sd)	4.44 (0.87)	4.44 (0.87)	2.16 (1.03)	6.88 (1.13)	5.68 (1.07)	4.60 (1.35)	6.16 (1.205)	4.92 (1.08)	4.41 (0.92)	4.48 (0.87)	4.28 (0.84)	2.31 (1.11)
	*F* value within study gr.	50.57*			22.94*			39.10*			39.62*		
	*F* value between study gr.										34.40*		

Collagen ratio (I/III)		1.25			1.57			1.49			1.31		

*P value <.0001, N-Normal skin, B-Before intervention of honey, A_15_-15 days after intervention and A_22_-22 days after intervention.

**Table 2:** Relative intensity distribution of E-cadherin expression in stratum basilaris and stratum spinosum.

Sample	Stratum basilaris		Stratum spinosum	
	Overall intensity distribution	Expression intensity according to cellular sites	Overall intensity distribution	Expression intensity according to cellular sites
Normal	8.40 ± 0.81	6.60 ± 0.57	7.68 ± 1.02	8.48 ± 0.51
Preintervention	3.96 ± 1.45	1.36 ± 0.49	3.72 ± 1.13	3.04 ± 0.89
15th day of Postintervention	6.80 ± 1.00	2.88 ± 0.88	6.26 ± 0.91	5.56 ± 0.92
20th day of postintervention	7.68 ± 0.80	6.00 ± 0.76	7.36 ± 1.11	7.84 ± 0.69
